# *Schistosoma* *mansoni* Infection in Preschool-Aged Children: Development of Immunoglobulin E and Immunoglobulin G_4_ Responses to Parasite Allergen-Like Proteins

**DOI:** 10.1093/infdis/jis676

**Published:** 2012-11-02

**Authors:** Angela Pinot de Moira, Jose C. Sousa-Figueiredo, Frances M. Jones, Colin M. Fitzsimmons, Martha Betson, Narcis B. Kabatereine, J. Russell Stothard, David W. Dunne

**Affiliations:** 1 Department of Pathology, University of Cambridge, United Kingdom; 2 Department of Infectious and Tropical Diseases, London School of Hygiene and Tropical Medicine, United Kingdom; 3 Disease Control Strategy Group, Liverpool School of Tropical Medicine, United Kingdom; 4 Vector Control Division, Ministry of Health, Kampala, Uganda

**Keywords:** IgE, IgG_4_, schistosomiasis, *Schistosoma mansoni*, preschool-aged children, desensitization

## Abstract

Specific immunoglobulin E (IgE) responses are upregulated during chronic schistosome infection and during allergy. These responses are tightly regulated during schistosomiasis. We have previously shown that IgE regulation depends on the extent and length of exposure to individual parasite allergen-like proteins. Here we compare the development of IgE and immunoglobulin G4 (IgG_4_) responses to the differentially expressed allergen-like proteins SmTAL1 and SmTAL2 among preschool-aged children from 2 villages with different levels of *Schistosoma mansoni* transmission. We found a lack of SmTAL1 responsiveness among all children, but evidence for IgG_4_-dependent IgE-SmTAL2 desensitization in both villages, occurring earlier among children from the village where the level of transmission was greater. Findings provide insights into the development and regulation of allergic-type immune responses.

Evidence is accumulating that preschool-aged children (PSAC) are at significant risk of schistosomiasis [1]. However, relatively little is known about the immunoepidemiology of *Schistosoma* species infection among these children and, hence, about the early development and regulation of the immune response to schistosomiasis in populations where *Schistosoma* species are endemic. Among older children and adults, chronic infection is associated with a skewed type 2 response, with elevated levels of specific immunoglobulin E (IgE) and eosinophilia [2]; these responses are also typical of allergy. In allergy, specific IgE induces a potentially lethal inflammatory response. A similar IgE response directed at antigen from relatively short-lived eggs that are trapped in host tissues everyday during schistosome infection [3] would be disastrous for both host and parasite. Instead, both have coevolved to produce/induce a tightly regulated immune response during infection, mediated by factors such as interleukin 10 and T-regulatory cells (Tregs), as well as immunoglobulin G_4_ (IgG_4_), which is capable of blocking IgE-allergen interaction [2].

We have shown previously that IgE regulation depends on the extent and length of exposure to individual parasite allergen-like proteins (Jones et al, unpublished data). IgE responses to SmTAL2, a member of the tegumental allergen-like (TAL) family expressed throughout the parasite's life cycle, including the egg stage [4], were low among long-term residents of a *Schistosoma mansoni*–endemic area of Kenya but significantly higher among recent immigrants to the same area. In contrast, SmTAL2-IgG_4_ responses were higher among residents; removal of IgG from sera resulted in significantly higher SmTAL2-IgE levels among residents, to the extent that levels were higher than those detected in immigrants. This demonstrates IgG-dependent desensitization of SmTAL2-IgE responses among individuals with long-term exposure.

SmTAL1 is another TAL protein but is principally expressed in adult worms; anti-SmTAL1 IgE is associated with immunity to infection [5, 6]. In the same study in Kenya, SmTAL1-IgE and SmTAL1-IgG_4_ levels were both high among residents and significantly lower among immigrants (Jones et al, unpublished data). In communities of endemicity, SmTAL1-IgE and SmTAL1-IgG_4_ responses increase with age and after chemotherapeutic drug treatment [4]. In the mouse, where schistosome worms outlive their host, SmTAL1-IgE responses only develop following repeated rounds of infection and praziquantel treatment, whereas SmTAL2-IgG and SmTAL2-IgE are seen relatively early (Jones et al, unpublished data). Taken together, this evidence suggests that SmTAL1 responses and associated immunity take much longer to develop after repeated exposure to dying worms.

In the current study, we investigate the development of IgE and IgG_4_ responses to SmTAL1 and SmTAL2 in PSAC. The study was conducted in 2 separate villages with different degrees of transmission. We compare age-related changes in IgE and IgG_4_ responses to SmTAL1 and SmTAL2, to determine how the extent of exposure determines the early development and regulation of these allergic-type responses. Previous findings would predict that few PSAC have anti-TAL1 responses, but that they might have greater, un-regulated, and potentially damaging, IgE-SmTAL2 levels. This combination of responses could result in increased susceptibility to infection and morbidity, highlighting the potential benefits of including PSAC in schistosomiasis control programs.

## METHODS

This study forms part of a larger Schistosomiasis in Mothers and Infants (SIMI) project which was conducted in 6 *S. mansoni*–endemic communities in Uganda and described in detail elsewhere [7]. The London School of Hygiene and Topical Medicine and the Ugandan National Council of Science and Technology granted ethics approval. Briefly, mothers and up to 2 of their children (age, 0.5–5 years) were recruited, and written informed consent obtained on behalf of children. Stool samples were obtained from each child on 2 consecutive days, and two 41.7 mg Kato-Katz thick slides [8] were prepared from each specimen; 75-µL blood samples were obtained by finger prick. Mothers were interviewed in the local language about their knowledge of schistosomiasis, their demographic characteristics, and both their and their children's water contact behavior and history of schistosomiasis treatment. The current study draws on baseline data and sera collected in April 2009 from 426 children of 213 mothers living in the villages of Bugoigo and Piida, Bulissa District, Lake Albert.

SmTAL1 (Sm22.6; XP_002575844) and SmTAL2 (Sm21.7; XP_002569898) were prepared as previously described [5]. Serum from blood samples obtained by finger prick was stored at −80°C until required. Levels of IgE and IgG4 to SmTAL1 and SmTAL2 were measured using biotinylated isotype-specific monoclonal antibodies, as described elsewhere [4]. Sample sera and plasma from noninfected European controls were assayed in duplicate at concentrations of 1:20 (IgE) and 1:200 (IgG_4_). A 3-fold serial dilution of purified human IgG4 (Sigma-Aldrich, United States) or IgE myeloma (Calbiochem, Germany) was added to each plate, forming a 14-point standard curve, starting at 30 µg/mL. Plates were read at dual wavelengths (490 and 630 nm) on a Powerwave HT microplate reader (BioTek Instruments). Results were interpolated from standard curves with a 5 parameter curve fit, using Gen5 analysis software (BioTek Instruments).

For analysis, infection intensity was expressed as mean egg count per gram (epg); geometric means were calculated to allow for skewness of data. Detection thresholds for enzyme-linked immunosorbent assay readings for each antigen and isotype were calculated as the mean plus 3 SDs of noninfected European control plasma samples. Risk factors for infection were examined using forward-fitting 2-level logistic regression analysis, to allow for correlations between siblings. Sex-adjusted associations between seroprevalence, age, and village were similarly examined using 2-level logistic models; age-village interactions were tested to determine whether associations varied with age and village. Nonlinear associations were examined by testing quadratic terms and categorical variables. Multilevel models were fitted in MLwiN (Bristol University, United Kingdom); other analyses were conducted using Stata, version 10.1 (StataCorp, United States).

## RESULTS

Overall, 42.1% of children had detectable *S. mansoni*, and the geometric mean infection intensity among those infected was 49.23 epg. The prevalence and intensity of infection varied significantly by village. In Bugoigo, the prevalence was 53.0%, compared with 27.5% in Piida (*P* < .001), and geometric mean intensity of infection among infected individuals was 61.38 epg in Bugoigo and 27.79 epg in Piida (*P* = .002).

The prevalence of key demographic and behavioral risk factors, determined by the questionnaire, is presented in Table 1 by village; also displayed are associations between risk factors and infection. The likelihood of infection was increased among certain ethnic groups, with age, with the duration of water contact, and on learning to swim (*P* ≤ .03). Children from Bugoigo were more likely to be of “other” ethnic groups (which was associated with a greater odds of infection), to spend more time in the water, and to be brought to the water by their mother, compared with children from Piida (Table 1); these behavioral differences help explain the higher prevalence of infection among Bugoigo children, although environmental factors are also likely to be important.

**Table 1.** Distribution of Risk Factors and Association Between Risk Factors and *Schistosoma mansoni* Infection Among Preschool-Aged Children (PSAC)

	Distribution^a^			Association With Infection		
Risk Factor	Bugoigo	Piida	*P*	PSAC Infected, %	Adjusted^b^ OR (95% CI)	*P*^c^
Village						
Bugoigo	…	…		53.02	Reference	
Piida	…	…		27.50	.20 (.09–.43)	<.0001
Sex						
Female	111 (51.15)	77 (47.83)		42.25	Reference	
Male	106 (48.85)	84 (52.17)	.52	41.71	1.15 (.60–2.20)	.66
Age, y, mean	3.11	2.94	.26		1.37 (1.06–1.76)	.02
Ethnic background						
Banyoro	18 (6.87)	14 (8.75)		41.38	.93 (.23–3.77)	
Bagungu	58 (22.14)	30 (18.75)		28.57	.31 (.12–.75)	
Alur	150 (57.25)	110 (68.75)		43.67	Reference	
Other^d^	36 (13.74)	6 (3.75)	.004	63.89	4.13 (1.03–16.62)	.004
Water contact duration, h						
Never	97 (45.12)	68 (42.77)		30.00	Reference	
<0.5	42 (19.53)	53 (33.33)		39.36	.76 (.25–2.29)	
0.5–1	27 (12.56)	21 (13.21)		55.32	3.06 (.90–10.45)	
>1 to 2	43 (20.00)	14 (8.81)		63.16	6.20 (1.71–22.50)	
>2	6 (2.79)	3 (1.89)	.01	77.78	6.78 (.34–134.69)	.01
Can swim						
No	185 (87.68)	111 (71.15)		39.45	Reference	
Yes	26 (12.32)	45 (28.85)	<.001	53.52	3.26 (1.14–9.33)	.03
Mother brings to water						
No	124 (65.26)	122 (80.26)		39.26	Reference	
Yes	66 (34.74)	30 (19.74)	.002	50.00	.43 (.16–1.17)	.10

Abbreviations: CI, confidence interval; OR, odds ratio.

^a^ Data are no. (%) of PSAC, unless otherwise indicated.

^b^ Estimated using forward-fitting 2-level logistic regression. The following variables were included and retained if significant at the *P* < .1 level: village, age, water contact duration, child treated for schistosomiasis, child can swim, ethnic background, mother brings child to water, site where child is bathed (lake vs home), frequency of bathing, mother's occupation, and whether mother had heard of schistosomiasis (or Bilharzia). Infection was defined as ≥1 detectable *S. mansoni* eggs in Kato-Katz slides.

^c^ By likelihood ratio tests.

^d^ Congolese and other minority ethnic groups.

**Figure 1.**
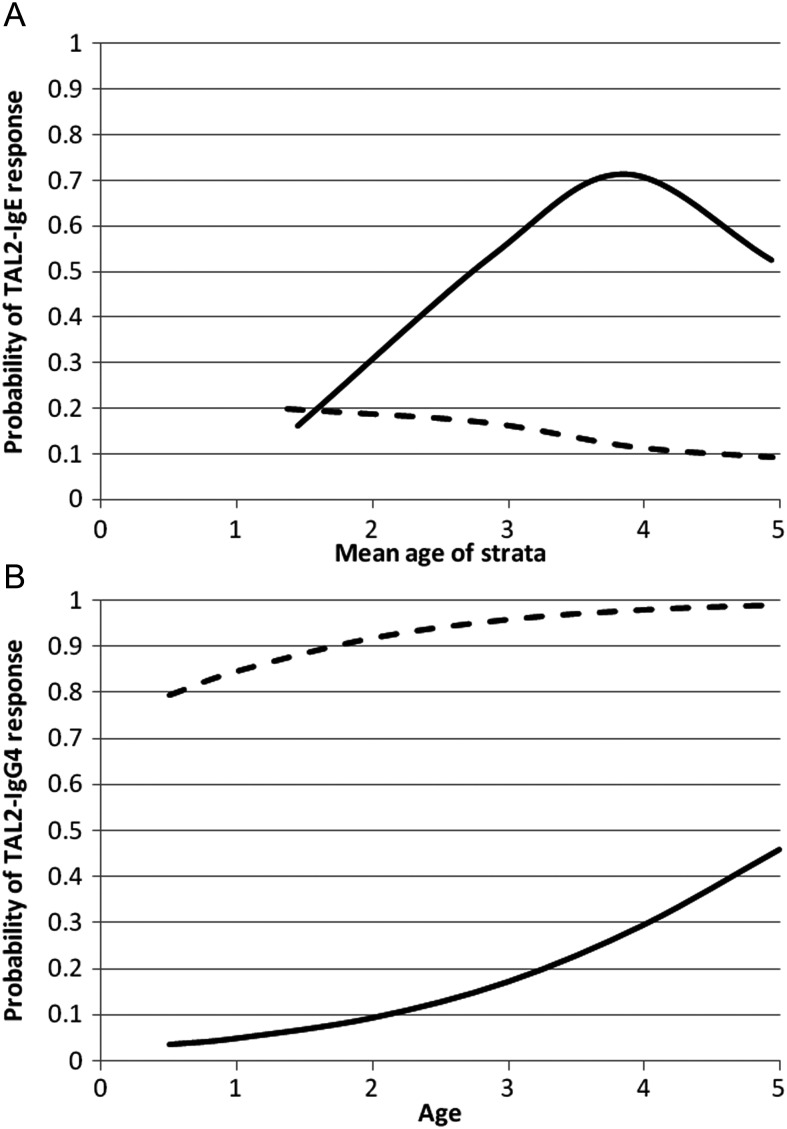
Predicted probability for TAL2 immunoglobulin E (*A*) and TAL2 immunoglobulin G_4_ (*B*) responsiveness among infants in Bugoigo (dashed line) and Piida (solid line). A total of 180 children in Bugoigo (68.7%) donated sera, 98 (56.0%) of whom had detectable infection. A total of 121 children in Piida (73.8%) donated sera, 37 (30.6%) of whom had detectable infection. *A*, A statistically significant age-village interaction was observed (χ^2^ [3 df] = 16.01; *P* = .001); age was modeled as a categorical variable because of departure from linearity for Piida estimates (*P* = .01). Model-predicted odds ratios (ORs) were as follows: male sex, 0.68 (95% confidence interval [CI], .36–1.29); village (Piida), 0.77 (95% CI, .26–2.32); age 2.1–3 years, 0.82 (95% CI, .27–2.55); age 3.1–4 years, 0.52 (95% CI, .14–1.97); age 4.1–6 years, 0.41 (95% CI, .11–1.54); age 2.1–3 years*Piida interaction term, 7.29 (95% CI, 1.33–40.05); 3.1–4 years*Piida interaction term, 25.36 (95% CI, 4.35–147.68); and age 4.1–6 years*Piida interaction term, 14.18 (95% CI, 2.43–82.76). *B*, No significant age-village interaction was observed (χ^2^ [3 df] = 0.374; *P* = .541); age was modeled as a continuous variable because there was no departure from linearity (*P* = .274). Model-predicted ORs were as follows: male sex, 0.44 (95% CI, .20–.94); village (Piida), 0.01 (95% CI, .003–.02); and age, 2.03 (95% CI, 1.54–2.67).

To investigate how the degree of exposure influences the early development of immune responses to *S. mansoni*, we measured children's anti-SmTAL1 and anti-SmTAL2 IgE and IgG_4_ responses. Virtually none of the 301 children who donated serum produced SmTAL1-IgE or IgG_4_ responses: SmTAL1-IgE and SmTAL1-IgG_4_ were detected in 1 child (age, 4 years) and in 2 children (mean age, 4 years; both were treated previously), respectively, at very low levels. In contrast, 72 (23.9%) children had detectable SmTAL2-IgE, and 180 (59.8%) children had detectable SmTAL2-IgG_4_. Although there was no significant difference in the prevalence of SmTAL2-IgE responsiveness among infected versus noninfected children (prevalence, 25.9% among infected children and 22.4% among noninfected children; *P* = .65 after adjustment for age and sex), the prevalence of SmTAL2-IgG_4_ responsiveness was significantly greater among infected children (prevalence, 72.6% vs 48.4%; *P* = .01 after adjustment for age and sex). The prevalence of both responses varied by village and with age; for anti-SmTAL2-IgE, associations with age varied significantly by village (age-village interaction, *P* = .001). Overall, 13.9% of children from Bugoigo had detectable SmTAL2-IgE responses, compared with 38.8% of children from Piida (*P* < .001 after adjustment for age and sex). Figure 1*A* displays the predicted probability of SmTAL2-IgE responsiveness over age, by village. Among infants from Piida, the predicted anti-SmTAL2-IgE prevalence initially increased rapidly with age but peaked and then declined at around 4 years of age. Among infants from Bugoigo, in contrast, the predicted probability was overall lower and decreased with age.

Figure 1*B* displays the predicted probability of an anti-SmTAL2-IgG_4_ response over age, by village. Unlike the predicted anti-SmTAL2-IgE prevalence, the predicted anti-SmTAL2-IgG_4_ prevalence increased linearly with age in both villages. Furthermore, the likelihood of a response was significantly greater among children from Bugoigo, compared with children from Piida (*P* < .0001 after adjustment for age and sex), with 89.4% of children from Bugoigo having a detectable SmTAL2-IgG_4_ response, compared with only 15.7% of children from Piida.

## DISCUSSION

SmTAL1 is a member of the TAL family, a family of proteins differentially expressed throughout the schistosome life cycle that share structural homology with the EF-hand allergens, one of the most common group of clinical allergens [4]. It is principally expressed in the adult worm and thought to be sequestered from the immune system in live worms. In areas of endemicity, responses to SmTAL1 steadily increase with age, it is thought following gradual, accumulated exposure to antigen released from dying worms [4]. SmTAL2, another TAL, is expressed throughout the parasite's life cycle, including the egg stage; hence, exposure is continuous during infection because of the release of SmTAL2 from short-lived eggs trapped in tissue. In contrast to SmTAL1-IgE, SmTAL2-IgE responses are low among long-term exposed individuals but significantly higher among recently exposed individuals; there is strong evidence to suggest that this is due to IgG_4_-dependent SmTAL2-IgE desensitization (Jones et al, unpublished data).

In the current study, we examined SmTAL1- and SmTAL2-IgE and IgG_4_ responses among PSAC from an *S. mansoni*–endemic region of Uganda*.* On the basis of findings from previous studies, we hypothesized that children would have no or low anti-TAL1 responses but higher, unregulated TAL2-IgE responses. The children studied were from 2 villages with different levels of transmission: children from Bugoigo had significantly greater risk of infection than children from Piida. In Bugoigo, SmTAL2-IgE responses decreased with age and were overall lower than in Piida, where responses increased then decreased with age. In contrast, the prevalence of SmTAL2-IgG_4_ responsiveness was higher in Bugoigo, and the likelihood of a response increased with age in both villages. These findings are consistent with previous observations comparing SmTAL2 responses among resident and immigrant populations (Jones et al, unpublished data) and provide further support for our hypothesis that SmTAL2-IgE is an early human immune response to *S. mansoni*, which is downregulated during chronic infection, probably because of IgG_4_-dependent desensitization. The rapid SmTAL2-IgE desensitization observed in Bugoigo highlights the acute nature of this response. Since the average lifespan of *S. mansoni* adult worms is 7 years [9], the observed lack of SmTAL1 responsiveness among PSAC is entirely expected and confirms that this is a much later response that develops after repeated exposure to antigen following natural or induced worm death.

Chronic schistosomiasis morbidity is caused by T-helper 2 granulomatous responses to continuous deposition of eggs, which over years [10] can cause severe fibrotic disease [11]. Acute schistosomiasis is also thought to be a reaction provoked by eggs, as well as by migrating schistosomulae [12]. IgE-mediated inflammation, triggered by egg allergen-like antigens such as SmTAL2, could play a role in this and could also occur in very young children in schistosomiasis-endemic areas. If so, SmTAL2-IgE modulation would limit IgE-mediated tissue damage, similarly to allergen-specific immunotherapy (SIT), in which repeated allergen administration is used to induce IgE desensitization. Immunological changes associated with SIT include reductions in IgE, induction of Tregs, and increases in allergen-specific IgG, particularly IgG_4_ [13]. IgG is thought to directly compete for the same epitopes as IgE, downmodulating both IgE-dependent histamine release [14] and IgE-facilitated allergen presentation to T cells [15].

In summary, the current study investigated the development of IgE and IgG_4_ responses to the allergen-like proteins SmTAL1 and SmTAL2 among PSAC from 2 separate villages with different degrees of *S. mansoni* transmission. We provided evidence for IgG_4_-dependent IgE desensitization to constitutively expressed SmTAL2; this desensitization occurred earlier in the higher transmission village. Almost no children had developed detectable responses to the worm antigen SmTAL1, most likely because of a lack of sufficient exposure to antigen. Our results confirm previous findings suggesting that the degree of IgE regulation is dependent on the extent and length of antigen exposure: we hypothesize that potentially pathogenic IgE responses to continuously-released SmTAL2 are tightly regulated among adults in regions of endemicity but that SmTAL1-IgE responses are less regulated, because of only periodic exposure following worm death. Findings will help our understanding of immune responses in schistosomiasis and in allergy, providing insights for the therapeutic treatment of both. A lack of immunity, combined with higher prevalence of pathogenic IgE responses, could increase the risk of severe morbidity among PSAC, highlighting the benefit for their inclusion in schistosomiasis control programs.
